# A Planar Millimeter-Wave Resonator-Array to Sense the Permittivity of COP Film with the 5G Handset Back-Cover

**DOI:** 10.3390/s21134316

**Published:** 2021-06-24

**Authors:** Yejune Seo, Changhyeong Lee, Inyeol Moon, Koichro Ota, Ryomei Omote, Sungtek Kahng

**Affiliations:** 1 Department of Information & Telecommunication Engineering, Incheon National University, Incheon 22012, Korea; M.June@inu.ac.kr; 2 Center for Advanced Meta-Materials, Korea Institute of Machinery & Materials, Daejeon 34103, Korea; Antman@kimm.re.kr; 3 Global R&D Center, NISSHA Korea, Inc., Seongnam 13591, Korea; iy-moon@nissha.com; 4 NISSHA Co., Ltd., Kyoto 604-8551, Japan; k-ota@nissha.com (K.O.); r-omote@nissha.com (R.O.)

**Keywords:** permittivity, measurement, film, 5G mobile handset, meta-material

## Abstract

In this paper, a new sensor is developed to estimate the dielectric constant of Cyclo Olefin Polymer (COP) film utilizable for 5G mobile phones’ multi-layered back-cover. It is featured by the electrical characterization of the thin layer of the COP film at 28 GHz as the material under test (MUT) directly contacting the planar probe (which is an array of resonating patches) and a new meta-surface as metal patterned on the COP film inserted between the planar probe and the 5G multi-layered back-cover for enhanced physical interpretation of the data by way of impedance matching. In this approach to delving into the material, a thin and small meta-surface film with an area of 25.65 × 21.06 mm^2^ and a thickness of 55 μm is examined for applications to 5G mobile 28 GHz-frequency communication on the basis of the below −10 dB-impedance matching for the 1-by-4 array sensor. Along with this, the real and commercial handset back-cover is brought to the test. The proposed method presents the advantages of geometrical adequacy to the realistic 5G handset antenna configuration, the idea of impedance-matching via meta-materials, and the suitability of characterizing the film-type structure as compared to the open-ended coaxial waveguide, waveguide-to-waveguide and TX horn-to-RX horn free-space test methods.

## 1. Introduction

Hyper connectivity has become a slogan of the 5th generation of mobile communication and data transmission is being maximized as a collective effort from a variety of wireless technologies (such as the broad-band, beamforming, carrier aggregation, and so forth [1]). While carrier aggregation is related to spectrum, broad-band and beamforming fall into the category of antennas. As hardware essential to the communication system (such as a handset and a base-station radio-head), an antenna links the transmitter with the receiver electro-magnetically. The functions of the antenna will be definitely influenced by the frequency, geometry and materials.

As 5G mobile communication adopts millimeter-wave bands (including 28 GHz) in order to realize the broad-band, the antenna is more sensitive to its geometry and materials inside and close to the structure, and its design becomes even more complicated [2]. During the design stage, an EM-field program is almost always used for accurate analysis, which necessitates *ε*_r_ as electrical properties of the constituent materials. The permittivity of each of the materials should be given from the supplier or figured out for full-wave EM simulation. As it was only recently that the 5G frequency bands were unveiled and the material vendors’ data mostly covered below 10 GHz, the antenna designer has to cope with sensing the permittivity of individual materials. Even if the vendor shares the material values at 28 GHz with the customer (since they are obtained from ideal setups that do not mimic interactions with close by layers or realistic EM-field sources), the antenna designer cannot avoid taking responsibility for estimating the permittivity of the target material by considering the real information on the antenna geometry, propagating modes and EM-coupling from layers. This enables the antenna designer to cut down on technical and functional errors.

In the field of dielectric–constant (*ε*_r_) extraction, the following test methods have been practiced quite commonly. They are classified as the open-end coaxial cable, the material-embedded waveguide cavity, and the free-space test with horn antennas. J. Mosig et al. put the open-end of a coaxial line contacting a dielectric material with an unknown permittivity. After setting up modal analysis equations based on the boundary conditions, they presented the material value from 1 GHz to 10 GHz, which ended up with errors from the approximations with the TEM fields, equivalent source and mode truncation [3]. J. Grant et al. called their test device a sensor and used it to find the dielectric constant of deionized water using an equivalent-circuit modelling backed by the Cole–Cole plot [4]. Liquid was handled with complex permittivity and they expressed the fringing field with the load admittance, which is an improvement from the TEM-mode approach. Their calculation is limited to 3 GHz and lower. G. Otto et al. showed dielectric measurement combined with the test calibration [5]. The test structure ended with a wide shorting cavity in which methanol or tap water was contained. The values were plotted up to 1 GHz with the modal analysis. This technique is not applicable to thin materials and materials placed in an open space. On the contrary to the first two schemes, D. Blackham et al. used a disk-loaded coaxial cable, which faces wider samples [6]. Based on the admittance from the modal expansion, they drew the curves of the complex *ε*_r_ of dissolved solids up to 20 GHz. Their scheme dealt with fitting the measurement curves. The device is not proper for thin dielectrics and requires the fabrication of a thick waveguide flange and connection to the coaxial line. Apart from those asymmetric geometries, materials under test (MUT) were put at the center of waveguide cavities as symmetric shapes [7,8,9,10,11]. Y. Wang et al. injected propyl alcohol or ethyl alcohol as the MUTs into the liquid container section between the waveguide ports [7]. They calculated the permittivity of the materials using the attenuation constant and propagation constant from the reflection coefficient, which holds only for the TE_10_-mode of a 12 GHz-band. A very similar experiment was conducted by M. Akhtar [8]. Epoxy resins were investigated, being inserted in WR-340. YIG (as nano-Ferrites) were put in between the waveguide sections by M. Al-Moayed et al. [9]. They adopted the Weir’s scheme to plot the constitutive parameters from 4.0 GHz to 5.8 GHz. However, all of their setups cannot deal with thin materials. U. Hasar et al. estimated *ε*_r_ by using the wave cascading matrices to convert s-parameters to the permittivity [10]. A. Kik solved the problem of *ε*_r_ of a sample by resorting to the modal series expansion and perturbation scheme [11]. This can give relatively accurate results, but the shortcomings stem from the stick-type MUT, the small container space of the ridge waveguide and TE modes not proper for handset antennas. The MUT is different from deriving *ε*_r_ from s-parameters of mismatched terminations in the open-ended coaxial cable and perturbation in a metallic cavity and is laid on the propagation axis from the TX horn antenna to the receiver horn [12,13,14,15,16]. G. Friedsam et al. illuminated the electromagnetic beam of the TX horn on the sample and investigated the transmission coefficient at the RX horn [12]. The TX beam should pass the convex lens held in the free-space, which would incur a high cost on the experiment. They related the tilting angle to the dielectric constant embedded in the transmission coefficient. N. Gagnon et al. built a setup the same as the free-space transmission scheme, but mentioned generating a Gaussian beam through the lenses [13]. The quality of the experiment is highly sensitive to the alignment between the setup elements, the gap between a lens and horn antenna, the gap between the lens and the MUT and the longitudinal symmetry. M. Afsar extended the Ka-band to Q-, V- and W-bands with bigger lenses [14]. This scheme might turn out to be costly due to the cautious shaping of curvy lenses and high-power sources. It looks like setting up an optical transmission test. C. Orlob et al. applied the line–network–network calibration technique to measurement configuration [15]. It is obligatory to generate the TX beam to satisfy the prescribed distribution of the electrical field on the sample. To lower the uncertainty stemming from misalignment of the line between the two horns, Y. Yamaguchi et al. replaced the RX part by the metal plate on which the material sample is put [16]. Unlike other free-space test schemes, they used the reflection coefficient without the transmission coefficient. Alongside this, there must be numerous modified versions of the representative practices to cope with constraints in the experiment (such as the physical shape of the material, the frequency and bandwidth of interest, the field intensity and distance, the kind of computation method, etc). These techniques can be classified in the following table (Table 1).

When it comes to material values needed in the 5G wireless antenna designs [17,18,19,20,21,22], the vendors using those methods should undergo large-scale changes.

A novel sensor is devised to get information on the dielectric constant of flat and flexible COP film at the 28 GHz frequency band glued to the 5G handset back-cover. Electronic circuits and modules embed Copper patterns on COP film as a signal line or a coupling path. It is valuable to check *ε*_r_ (the real part of its permittivity) and take the constant into account in the design of a component or an antenna enclosed by the back-cover. Inside the handset (since COP film is flat like the MUT), the sensor is desired to be correspondingly flat, which cannot be realized by the open-ended coaxial cable, waveguide cavity or horn-antenna included in the free-space test method. Alongside this, considering metal patterned COP film to be used inside a mobile phone and its size (such as in FPCB parts longer than 1 cm and shorter than 7 cm), it is reasonable to set the sensor as long as 5 cm. Therefore, the sensor (which should have a resonator for high sensitivity) becomes a 1-by-4 resonator array with reference to the half-wavelength of 28 GHz. This sensor touches the COP film on which the back-cover layers as an extended MUT. Different from the conventional permittivity sensing techniques where unpredictable reflection occurs and ends up with increase of the uncertainty in estimation, structural impedance matching is conducted from the RF input of the sensor all the way to the end of the extended MUT by way of a certain kind of 6μm-thick metal pattern on the COP film (55 μm-thick) as a meta-material. We fabricated this through NISSHA’s film processor, and the scattering parameter S_11_ of the prototype is measured. The measurement data is compared with the simulation data of the same structure with varying *ε*_r_ of the COP in the EM-field analysis program. After the iterative comparative observation, the estimation procedures are terminated when the work-flow satisfies the conditions of the lowest reflection coefficient below −10 dB and the smallest discrepancy between the measured and simulated S_11_. That is when *ε*_r_ is found. This novel approach results in the by-product information that the properties of the layers above the COP film within the back-cover are verified. In this paper, the behavior of the MUT is kept track of at the 28 GHz-band and will be applicable to other millimeter-wave frequency bands. This research work is organized in the following manner: a display of a 5G handset with the back-cover covering essential RF components, the back-cover as a stack of layers and the conventional test schemes for an MUT of interest in Section 2, the proposed measurement configuration with a novel sensor consisting of an array of 28 GHz resonators and a novel meta-surface for impedance matching in Section 3, the test results of the fabricated prototype as well as the full-wave EM analysis Section 4, discussions on the positive characteristics of the new sensing methodology through a variety of comparisons for different perspectives of view in Section 5, and observations that there is no need to use a waveguide or horn antennas, which means this new technique is neither heavy nor bulky.

## 2. Shape of the Mobile Handset and Commonly Used Test Schemes for Material Sensing

### 2.1. A Sketch of the Back-Side and Lateral Sides of the 5G Handheld Device

**Figure 1:**
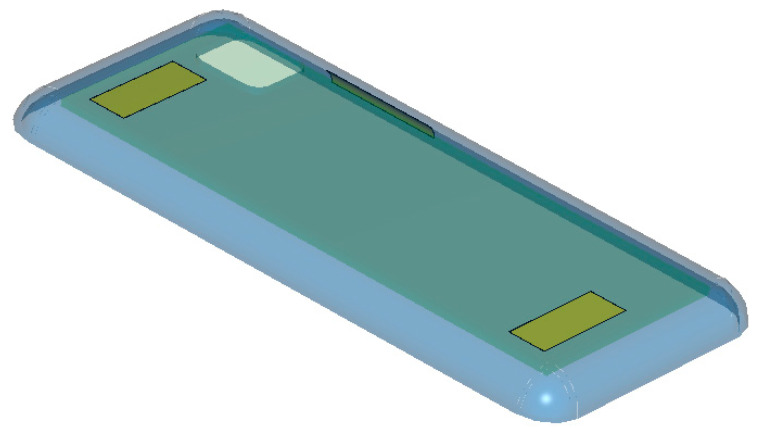
A bird’s eye-view of the in-fashion 5G mobile handset.

Before scrutinizing a particular part of the 5G handset (the so called high data-transmission smart phone), it needs to be viewed as a 3-dimensional structure as in Figure 1. As the numbers on width, length, height, etc. are given in Table 2, the device is as big as one’s palm.

Compared to the immediately past generations’ phones, the smart phone has outgrown the one that operated the service of lower data-transmission communication and smaller displaying. More functions and media have been plugged into this device, which drives it to be bigger and heavier.

Figure 1 is the 3D sketch of the 5G handheld device, which is easily seen in people’s daily lives. Specifically, its back-side is presented in line with the back-cover, the electrical attributes of which will be investigated through the novel sensor at a millimeter-wave frequency band. The back-side has been made larger than before to accommodate more optical lenses for an enhanced quality of photographs and biometrics authentication for security-sensitive transactions. Millimeter-wave antennas have been introduced to the layout of lower frequency antennas and the wireless power charging circuit block under the back-cover.

### 2.2. Conventional Measurement Schemes of Dielectric-Constant Extraction

**Figure 2:**
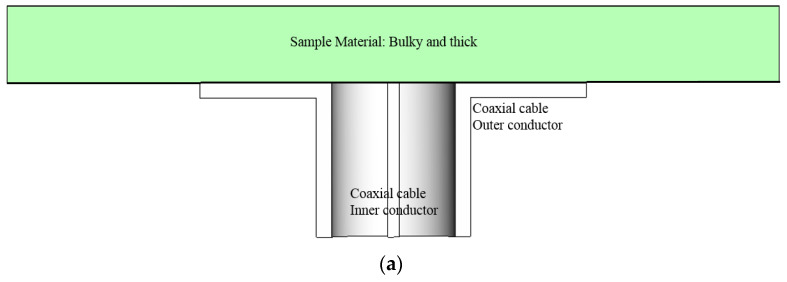

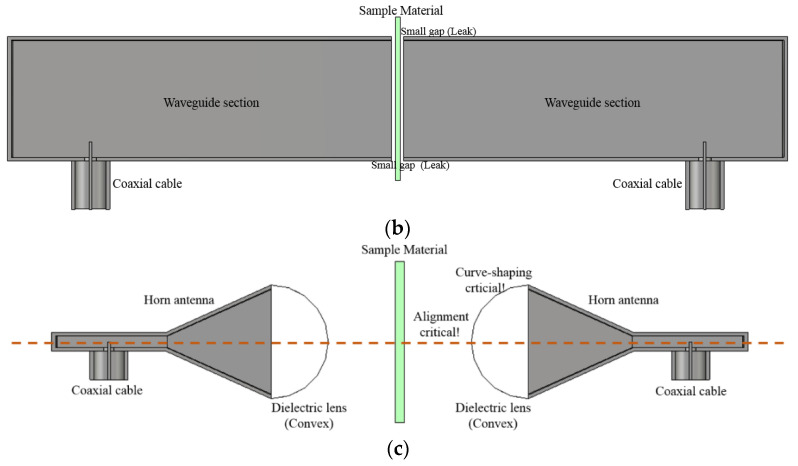
The most commonly used test setups to find the permittivity of a dielectric sample (**a**) Open-ended coaxial line (**b**) Embedding in a waveguide cavity (**c**) Line-of-sight link between horn antennas.

As was mentioned earlier, three measurement schemes are available in the industry for permittivity extraction of dielectric materials used in manufacturing electronic goods. The open-end of the coaxial line contacts a small spot on one side of a thick material. The open-end frequently flares to a flange, which ends up with a weight-increasing metallic attachment. Because this causes impedance mismatch to the coaxial cable and the TEM-mode field is distorted due to the heterogeneous cross-sections of the two sides, the conversion equation from the reflection coefficient to the permittivity tends to be less accurate, since the formula is based on transmission-line approximation and only one equation has a limited degree of freedom to the solution. Thin polymer dielectric materials have been studied and are characterized as almost real-valued permittivity, while thick dielectrics and liquids show complex permittivity, which runs across failure in complex *ε*_r_ extraction using the scheme above. This motivates us to carry out impedance matching for the prosed research work. Contrary to the reflection-coefficient-only measuring device, Figure 2b,c introduces the setups, enabling them to use both transmission and reflection coefficients. It should be pointed out that the last two setups lack impedance matching. Theoretically, a thin MUT is inserted between the two sections of a waveguide cavity, but gaps must occur between the metallic rim and the MUT or tight assembly will leave the thin material with wrinkles. The enlarged version MUT can be placed amid two horn antennas. The horn antennas are equipped with dielectric convex lenses, which require high precision grinding and are heavy and expensive. Failure in sensing the material permittivity is commonly caused by misalignment of the line-of-sight of the TX and RX horns and the inclination of the enlarged film sample. This gets worse as the operating frequency goes up. The last two methods are not appropriate for the development conditions of mobile phone modules. This spurs us to work on a novel approach to circumvent the above shortcomings.

## 3. Design of the Sensor: The Resonator Array and the Meta-Surface

The layers of the back-cover are of our concern, and the 3D geometry in Figure 1 is dissected along either of the principal axes into the side-view as the 2D information. Related to the contact-type measurement configuration for a flat material as in Figure 2a, the 2D views of the back-cover’s original shape and COP film loaded without or with a meta-surface are comparatively shown as follows.

**Figure 3:**
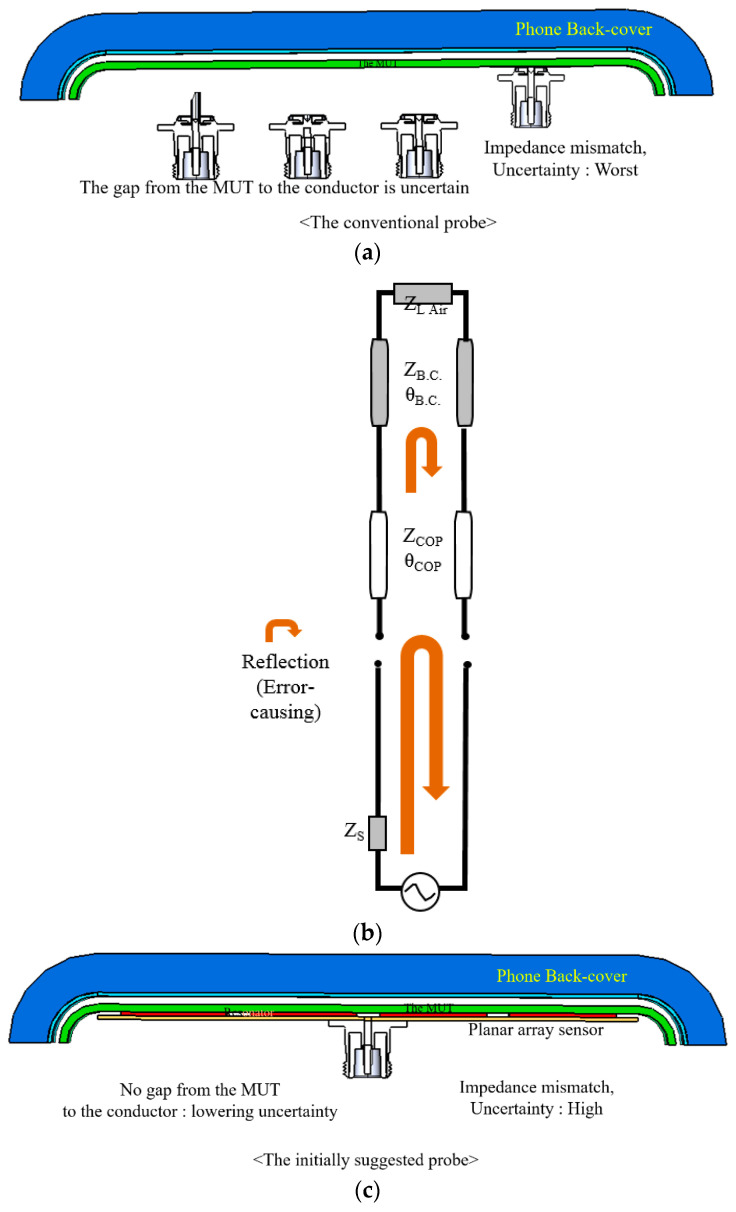

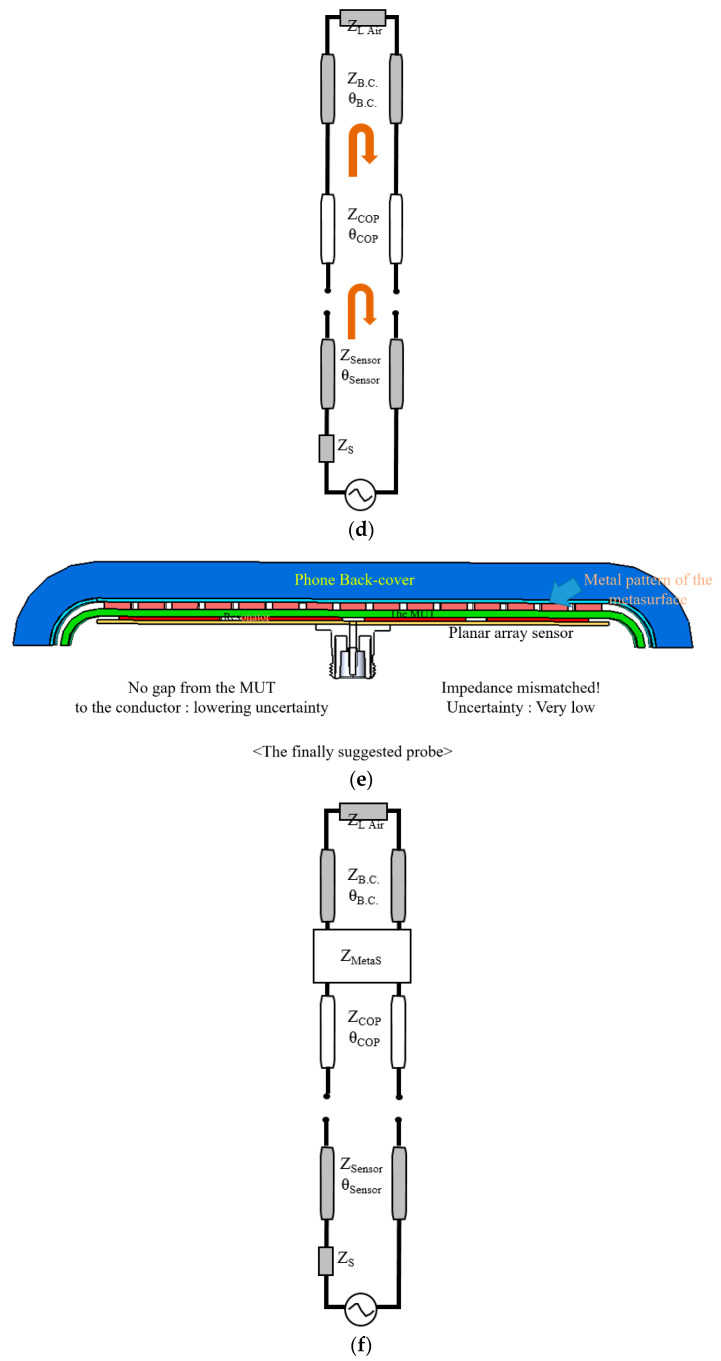
2D views of the sensor contacting the back-cover with changing conditions and their equivalent circuit models. (**a**) Original case. (**b**) Circuit model of (**a**). (**c**) Initially suggested probe. (**d**) Circuit model of (**c**). (**e**) Finally suggested probe. (**f**) Circuit model of (**e**).

Figure 3a shows the possible adaptation of the use of the open-ended coaxial cable in the permittivity extraction of layered media. The coaxial connector is slightly distant from the MUT, with the inner conductor sticking out, withdrawn from or ending at the plane of the flange, all of which are barely useful in this experiment. Imitating Figure 2a, the aperture of the coaxial cable touches the COP layer below the back-cover. In this case, the coaxial cable is not electrically compatible with the dielectric, and reflection severely occurs at the interface between the input transmission-line and the COP segment as shown in Figure 3b. There is another reflection from the mismatch between the COP film and the back-cover. The former reflection is mitigated by changing the direct contacting coaxial connector to a resonator array as mode transition from TEM to TM-mode as shown in Figure 3c,d. The latter reflection will be eased through the introduction of a meta-surface. As the initially suggested probe, sensitivity can be enhanced through Figure 3c. As a note, the equivalent circuit model is based on the transmission-line modelling each layer as the transmission-line segment of electrical length (*θ*_COP_) for the thickness and characteristic impedance (*Z*_COP_) for constitutive parameters [23,24,25,26]. As for Figure 3d, the voltage supply is connected to the cascading of transmission segments ((*θ*_Sensor_, *Z*_Sensor_), (*θ*_COP_, *Z*_COP_) and (*θ*_BC_, *Z*_BC_)) as the sensor, COP film and back-cover, respectively. There still exist reflected fields at the two interfaces. This reflection can be suppressed via structural impedance matching through the meta-surface denoted as *Z*_MetaS_ in Figure 3e,f. This is called the finally suggested probe, which requires a metal pattern to convert the COP film to work as a meta-material. Figure 3e reveals an extremely thin layer (2 oz thick) to bring down the reflection coefficient to the input or RF voltage supply as illustrated with return-signal arrows diminishing. The meta-material surface is used to match the impedance of material layers for the first time to our knowledge. It is expressed as the block remarked as *Z*_MetaS_. The lower S_11_ (the reflection coefficient), the more improvement in accuracy for the permittivity sensing. When an MUT is given, its electrical characterization is carried out by a process wherein a sensitive sensor or probe is developed, and if it is a 1-port test scheme, the reflection coefficient is checked and improved by a device to ease the local and cumulative impedance mismatch from the discontinuity planes between layers of the MUT and its surroundings.

**Figure 4:**
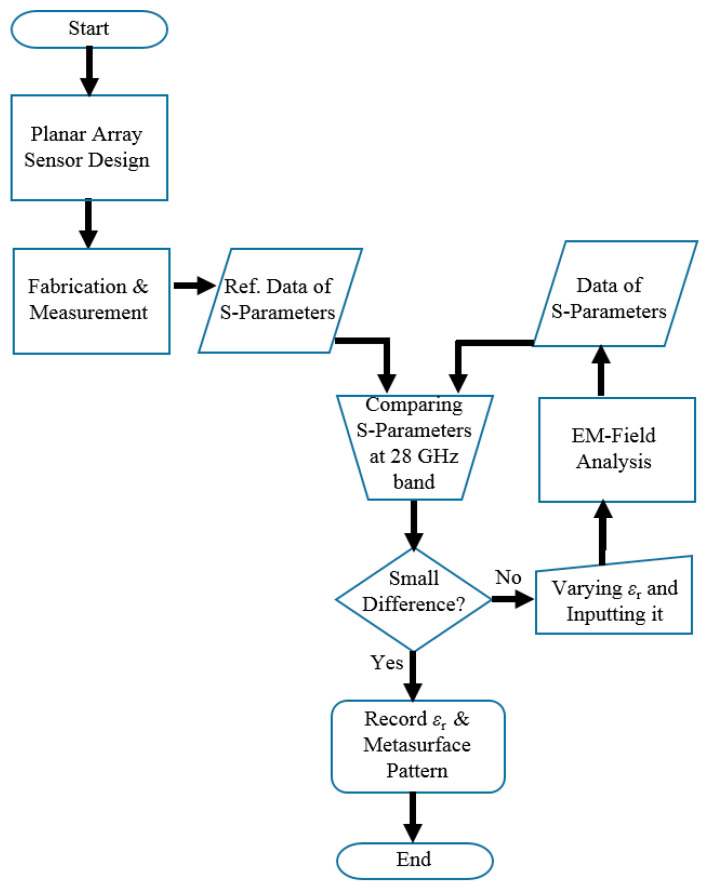
Flow chart of characterizing the permittivity of the material under test linked with the proposed impedance-matched sensor.

Figure 4 illustrates the workflow of how to find the electrical value of the MUT underneath the back-cover if and only if the novel sensor is employed. Reading the flow chart, the process is commenced with the design of a resonator array for sensing. This does not mean the millimeter-wave array alone but takes into account the entire structure and constituent materials, including the initially guessed *ε*_r_ for the MUT. Going over the information shared by the electronics industry on sub-strate dielectrics, different from thick or fluidic materials turning out to have complex *ε*_r_, the relative permittivity is dominantly real-valued and ranges from 2 to 5 for thin dielectrics such as CCL, OCA, MVD, COP and so on. The film is attached to the back-cover and touches the array sensor for manufacturing. This specimen is fabricated by NISSHA as the co-worker exclusive to this project, and they deliver the prototype to the laboratory. Measurement is conducted to have S-parameters, and the experimental data are compared with the EM simulation results with respect to the reflection coefficient over the 28 GHz-band. The EM simulation is run with the value of the relative permittivity ranging from 2 to 5, as mentioned above. This comparison results in a negligibly small error, which judges the guessed *ε*_r_, to meet the need. If the error is large, the process takes the observer to another round of EM simulation with a different relative dielectric constant. As the value converges, the process is terminated. This workflow is also utilized to shape the geometry of the meta-material surface pattern on the COP film, which is determined by watching the impedance matching for the whole sensor structure. A Fractal pattern belonging to the Sierpinski type is suggested to solve the guided wave and coupling problems with contacting stacked materials, unlike other meta-material design cases wherein the EM-field source is located away from the meta-surface with a large gap. This will be addressed in detail on the heels of talking about the array for RF signal excitation.

This sensor mainly works on the basis of an array of resonators fed by the RF-signal connector.

**Figure 5:**
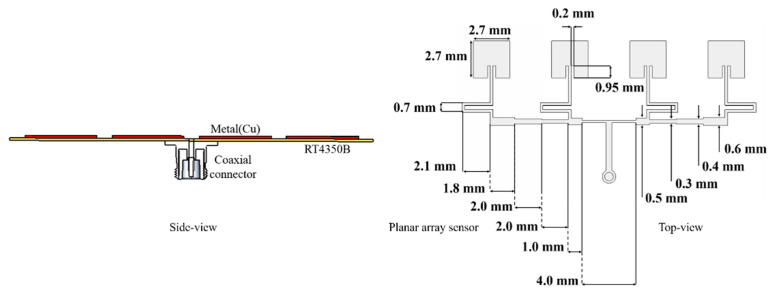
The resonator-array as the feed for the sensor: Side- and top-views of the geometry.

The geometry shows an array of rectangular patches (half-wavelength) connected through a transmission-line power-divider from the feed connector. While the coaxial cable goes well with the TEM-mode test, the novel sensor is suitable for the TM-mode, penetrating planar MUTs. In Figure 5, the size of the identical patches each is set as 2.7 mm-by-2.7 mm to resonate at 28 GHz, and they are copper of 18 μm-thickness patterned on RT4350B substrate (0.254 mm-thick). The other physical dimensions are connoted. As to the ordinary resonant sensor, it uses a single resonator, which is apt to a small sample. Nonetheless, there are four patches in this device to sense the EM fields over a spread and wide area of the MUT film, and they are assembled by the power-divider to collect distributed energy. The impedance of just the resonator array does not have to be perfectly matched now, and will be finally matched in a complete structure. This resonator array is yet to be loaded with material layers. First, the 5G back-cover, whose material and layering information is revealed in [23,24,25,26], is added.

The electronics industry adopts thin films (such as FPCB) inside plastic housings (such as the back-covers of mobile terminals), and the models here hold the two kinds with RF signal resonators. Catching the information [23,24,25,26], the back-cover is modelled with *h*_B.C._, *gap*_~Array_ and *ε*_r_, 827 μm, 58 μm, and 4.7 calculated by the mixing formula for layered dielectric media [25,27], comprised of Glass-, PET-, MVD/UV- and Silk-layers glued using OCA from top to bottom. Parameters *h*_COP_ and *gap*_~COP_ are 55 μm and 3 μm, respectively. The relative *ε* of the COP film is yet to be found, but is given initially as 2.0. The electrical properties peeked by the input impedance from the resonator array through the back-cover loading to the COP layer-combined back-cover are plotted as follows.

**Figure 6:**
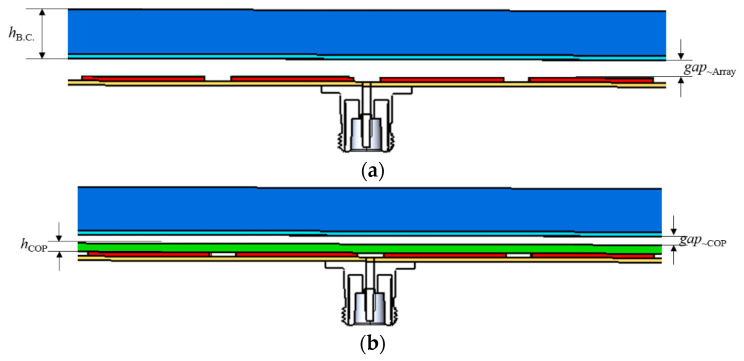
The loading of the back-cover and COP film with the resonator array. (**a**) Back-cover above the array. (**b**) Array contacting the COP film below the back-cover.

The proposed array sensor enables readers to watch the impedance of Figure 6a,b as the locations on the Smith chart. M0 is a good impedance match, as being the closest to the center of the chart, changes to M1 by the back-cover and M2 by COP film, getting worse. This can be improved with a meta-surface in that the following geometry is the unit cell and is extended to an array for coupling with the patch.

**Figure 7:**
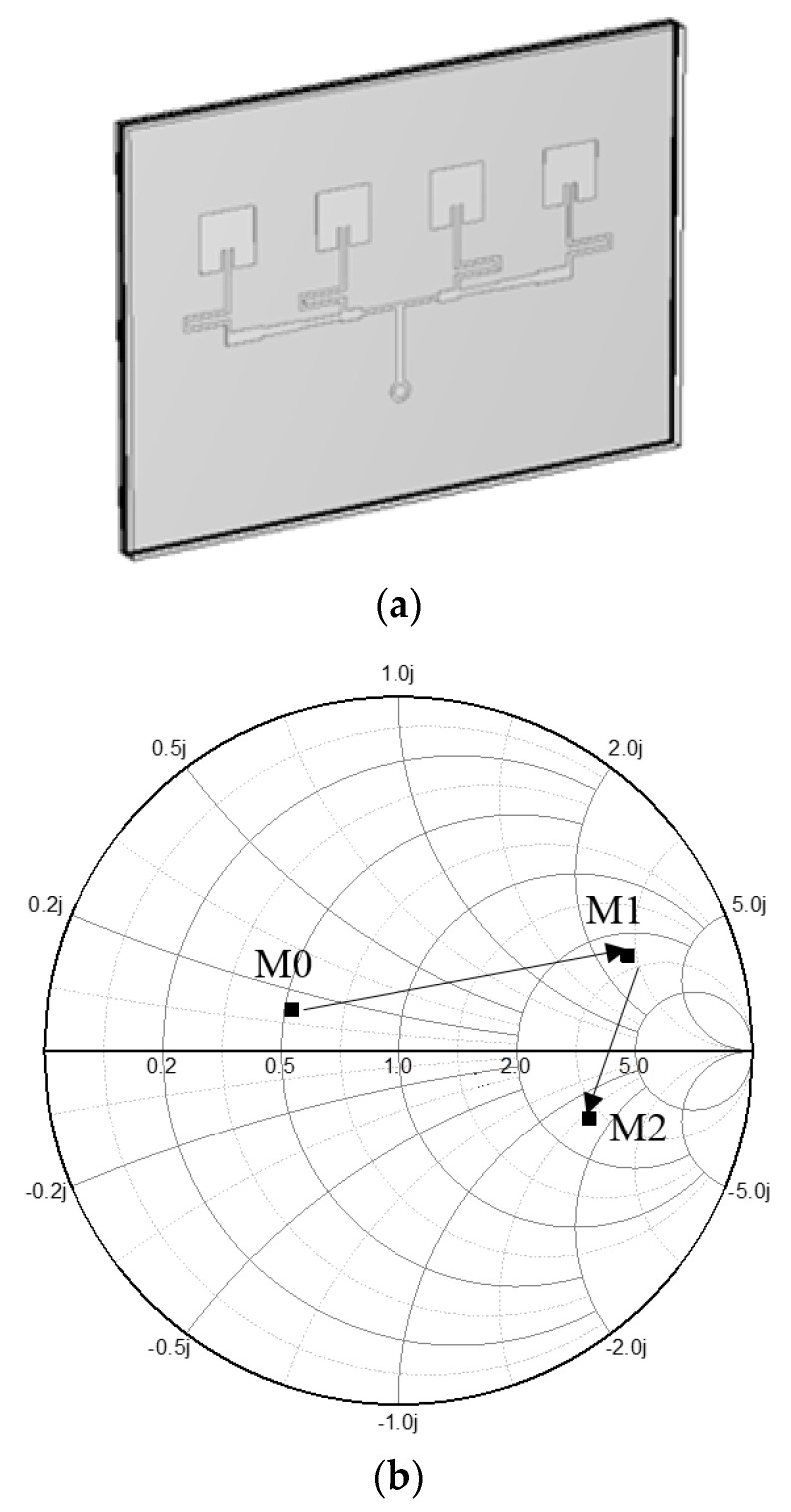
The Smith chart plot presenting the impedance of the resonator array: M0 as the array alone, M1 with the back-cover loaded and M2 with the COP film added. (**a**) Structure. (**b**) Smith chart.

**Figure 8:**
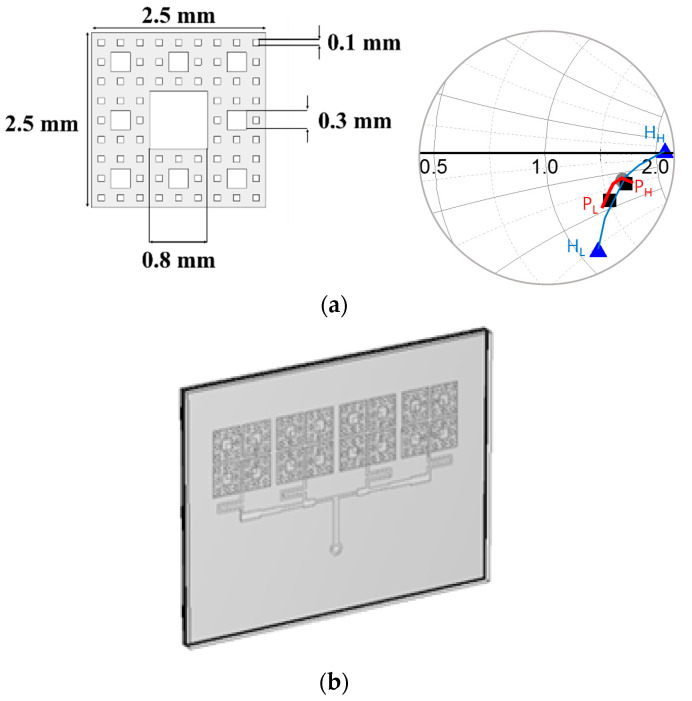

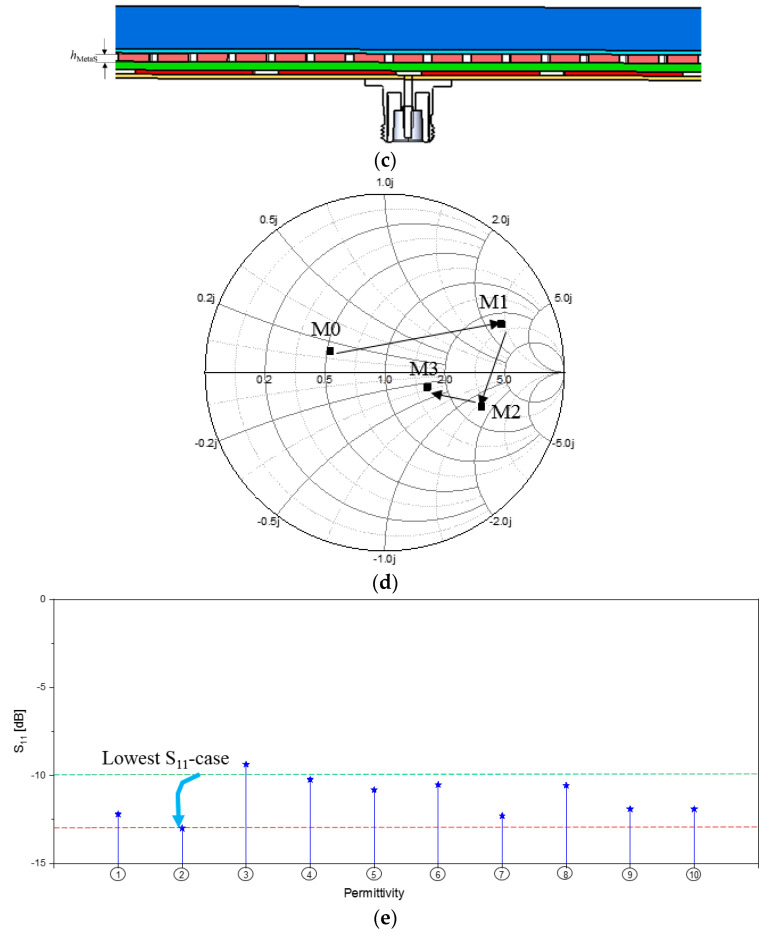
The input impedance of the sensor with a novel meta-surface (**a**) The unit-cell and its parametric study. (**b**) Meta-surface sandwiched by the COP film and back-cover. (**c**) Side-view. (**d**) Trace reaching the impedance matching. (**e**) Best guess is found as the permittivity of the COP film.

The impedance of the feed is enhanced as in Figure 7. Furthermore, it is noteworthy that the impedance matching is realized not by lumped elements but by a physical geometry. Most of all, attention must be drawn to the fact that the meta-surface physically contacts its upper and lower layers without a sufficient gap, not to mention a self-similarity metal pattern is used to solve a challenging subject of distributed-element impedance matching. A third-order of the Sierpinski Fractal structure is proposed with voids (or holes) on the metalized square in Figure 8a. The lower-order Sierpinski cells are so capacitive that they cannot move the impedance point close to the center of the Smith chart, but the third-order cell makes the impedance approach the center. To find the best set of geometrical parameters of the third-order Fractal structure, the parametric study was conducted with *P* as the length varied from *P*_L_ (2.30 mm) through *P*_M_ (2.50 mm) to *P*_H_ (2.70 mm), and *H* as the hole size varied from *H*_L_ (0.35 mm) through *H*_M_ (0.40 mm) to *H*_H_ (0.45 mm). The results of the parametric study reveal in Figure 8a that while *P*_L_, *P*_H_, *H*_L_ and *H*_H_ make their impedance points farther away from the center of the Smith chart, the combination of *P*_M_ and *H*_M_ work optimally for the impedance matching, as the Sierpinksi Fractal unit-cell is extended to a one-by-four array and covers the four resonators of the probing array. This optimized structure is interpreted electrically with the equivalent circuit models in the discussion. The resultant Fractal-array impedance-matching device is presented in Figure 8b. It is accompanied by its side-view in Figure 8c. The trail of the impedance-matching status is expressed in the Smith chart of Figure 8d where M0 is moved to M1 by the back-cover and M2 as the effect of the initially suggested probe and becomes M3, which accesses the center via the meta-surface. All these experiments are carried out with different choices of relative permittivity named ①~⑩. That is to say, the *ε*_r_ of ①, ②, ③, ④, ⑤, ⑥, ⑦, ⑧, ⑨, and ⑩ are given as 2.62, 2.30, 4.30, 3.80, 3.25, 3.50, 2.90, 3.66, 2.20 and 2.21, respectively. The choices result in the reflection coefficient of the sensor, and there are two criteria to justify the final choice. One is below −10 dB and the other is the lowest. These represent the best impedance match. In Figure 8e, choice 3 is ruled out from the candidates because the S_11_ is over −10 dB. Applying the second criterion to the rest of them, choice 2 meets the best condition due to it having the lowest S_11_. Therefore, the *ε*_r_ of the COP film is 2.3. This will be verified later by comparison with measurement.

## 4. Experimental Test on the Prototype of the Sensor

The resonator array and its meta-surface integrated version structure are fabricated, and their photographs are provided as follows.

**Figure 9:**
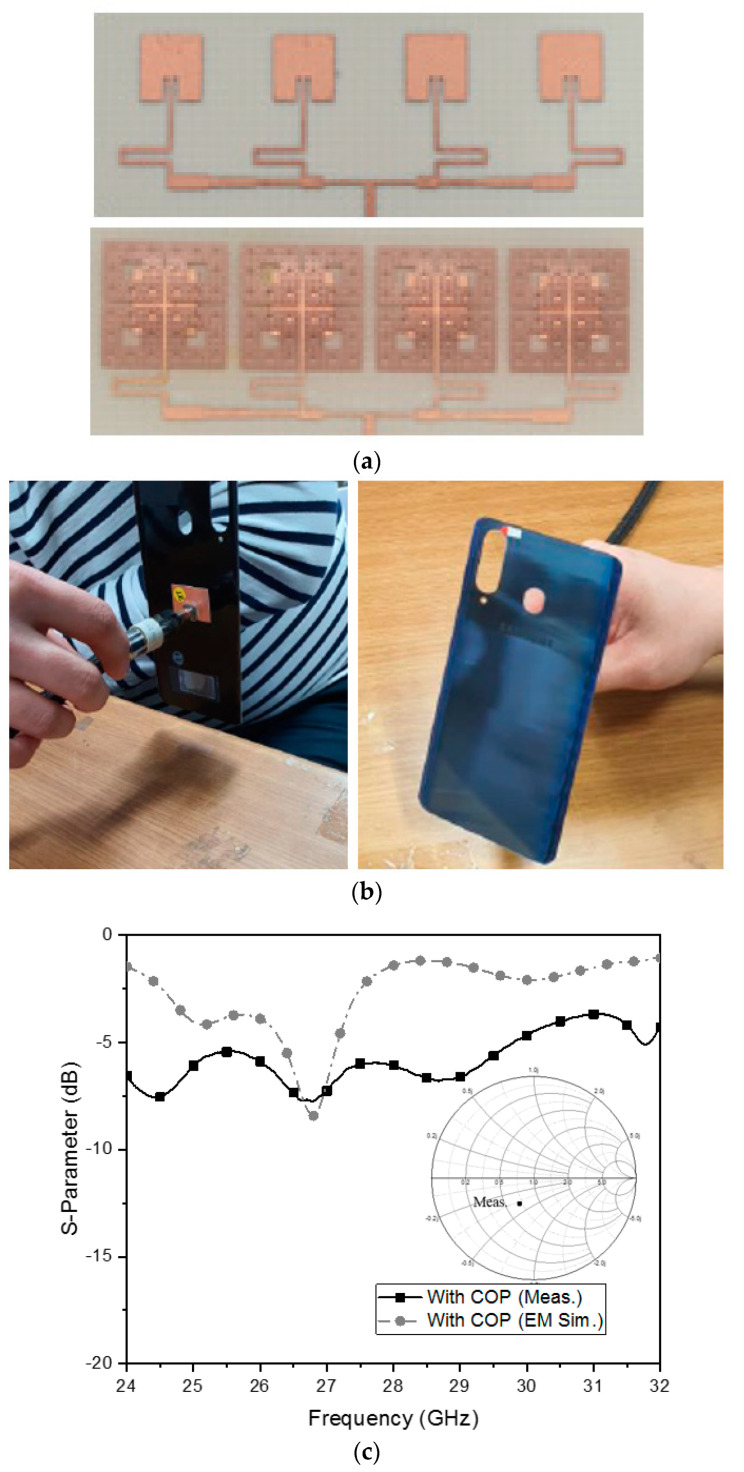

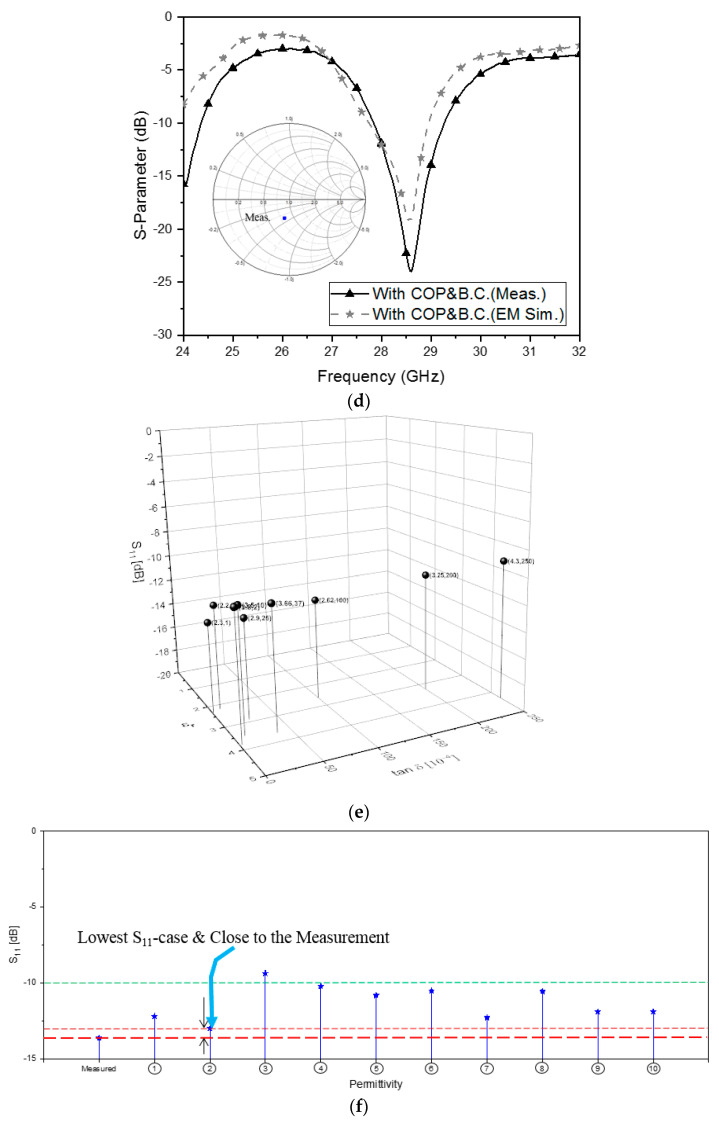
Physical realization of the proposed sensor and frequency responses. (**a**) The sensor before and after placing the meta-surface patterned COP film. (**b**) Proposed sensor attached to the back-cover. (**c**) S_11_ of the back-cover with the COP film. (**d**) S_11_ of the back-cover with the meta-surface COP film. (**e**) S_11_ of various choices in the permittivity plane. (**f**) S_11_ of various choices of permittivity.

Figure 9a contrasts the resonator–array sensor with the meta-surface- and COP-film loaded sensor as fabricated prototypes. The fractal cells of the meta-material optimized with voids on the metal squares and the gaps for lowering the capacitance between the COP film and the back-cover and giving inductance of surface current and parallel capacitance of the gap can move the M2 point to the M3 point in the Smith chart, which indicates the impedance matching. Figure 9b shows that the sensor in Figure 9a is attached to a real back-cover of a popular 5G handset. The input impedance of the proposed sensor lacking or having the meta-surface between the COP film and back-cover is measured and plotted as the insets of S_11_-curves in Figure 9c,d. The EM-simulated and measured results agree well with each other, to some degree. The former case has relatively poor impedance matching, as S_11_ is larger than −10 dB and the curve falls around 27 GHz. Meanwhile, the S_11_ of the latter case has a dip at 28 GHz below −10 dB (meaning the matched impedance). This leads to the finding of *ε*_r_ of the COP film through Figure 9e,f. Figure 9e is the cubic arrangement version of Figure 9f with the loss tangent added. In both graphical settings, the 2.3 value of *ε*_r_ as choice 2 has the lowest refection coefficient, with S_11_ below −10 dB (green line). When one more criterion (that the EM-simulated S_11_ should be as close as possible to the measured value at 28 GHz) is applied to the choices below the green line, choice 2 is located in the best vicinity of the measured reflection coefficient. So, the relative dielectric constant of the MUT turns out to be 2.3 by the results of this experiment.

## 5. Discussions

In this section, the characteristics of the proposed sensor are analyzed in terms of its electromagnetic and electrical aspects. Electrical engineers and material scientists must be wondering if there is a relationship between meta-surface based impedance matching for a layered structure [28], enhancement in electromagnetic field propagation and technical merits.

**Figure 10:**
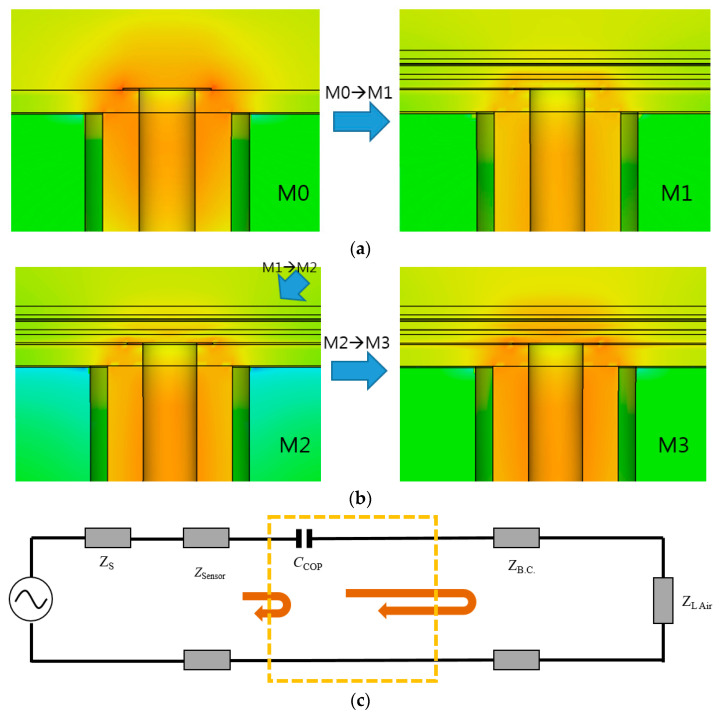

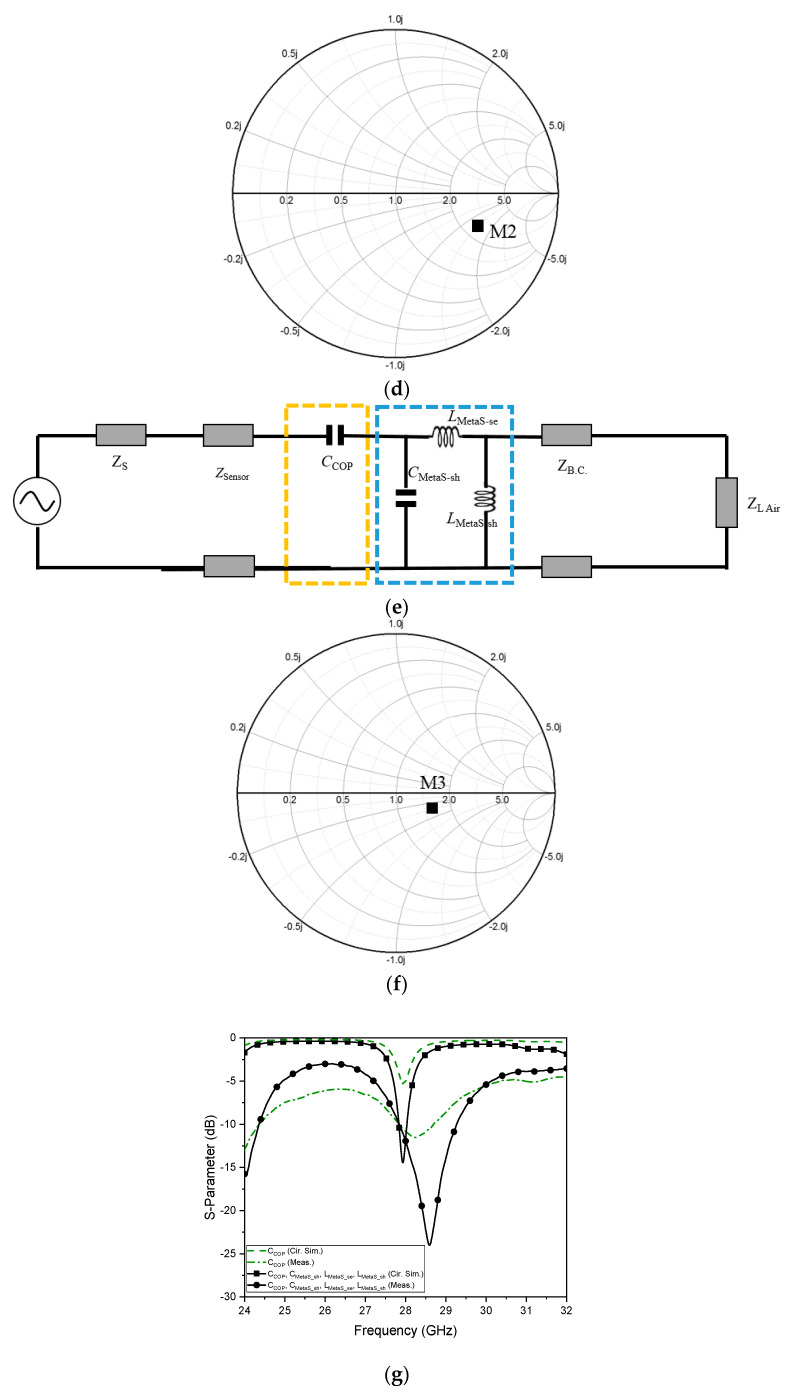
Electromagnetic-field and electrical circuit modelling points of view on the novel MUT sensor. (**a**) EM field of the array sensor alone is degraded by the back-cover and COP-film. (**b**) EM field becomes normal by the impedance matching via the meta-surface. (**c**) Electrical circuit model of the effects of the COP film. (**d**) Impedance of the capacitive effect of COP film. (**e**) Circuit model of the COP-film, meta-surface and back-cover together. (**f**) Impedance of the additional circuit block as the COP film meta-surface. (**g**) Comparing s-parameters of the circuit model analyses and the corresponding experiments.

Figure 10a,b present the change of the electric field-distribution of the sensor structure, with the successive addition of materials and variation in its corresponding input impedance. The array sensor alone (named M0) has a strong field, distributed to the farthest point. This is referred to as the reference. When the array sensor faces only the back-cover, the E-field becomes weaker (as M1). Impedance mismatch degrades the field strength. As the COP film is laid on the array sensor below the back-cover, the E-field is still weak (as M2). Nonetheless, by adding the meta-surface on the COP film, the E-field intensity becomes restored as seen as M3, which is enabled by desirable effects of impedance matching. Apart from the electromagnetic observations above, the approach taken to identify *ε*_r_ of an MUT can be understood with electrical circuit models. Figure 10c,d are the circuit model and its input impedance. M2 is obtained in a very similar manner to Figure 8, which means that the use of the COP film below the back-cover corresponds to a capacitive effect, expressed as a *C*_COP_ that is 0.027 pF. The crooked arrows of the signal in Figure 10c denote the reflection of the input signal due to the impedance mismatch. It can be mathematically figured out by the following formulation.

(1) $$\Gamma_{M1}=\frac{Z_{M1}-Z_{M0}}{Z_{M1}+Z_{M0}}$$

and

(2) $$Z_{M1}=\frac{A_{COP}}{C_{COP}}$$

where

(3) $$\left[\begin{array}{cc} A_{COP} & B_{COP}\\ C_{COP} & D_{COP} \end{array}\right]=\left[\begin{array}{cc} 1 & -j\frac{1}{\omega C_{COP}}\\ 0 & 1 \end{array}\right]\left[\begin{array}{cc} 1 & Z_{B.C.}\\ 0 & 1 \end{array}\right]$$

$\Gamma_{M1}$ is the reflection coefficient of the former circuit as a drawback, and is corrected by placing the meta-surface right next to *C*_COP_ as the composite form comprising *C*_MetaS_sh_, *L*_MetaS_se_ and *L*_MetaS_sh_, whose values are 0.027 pF, 0.381 nH and 3.000 nH, respectively, in Figure 10e,f. Like the previous circuit, an extended circuit model for $\Gamma_{M3}$ is solved with the following equations.

(4) $$\Gamma_{M3}=\frac{Z_{M3}-Z_{M0}}{Z_{M3}+Z_{M0}}$$

and

(5) $$Z_{M3}=\frac{A_{tot}}{C_{tot}}$$

where

(6) $$\left[\begin{array}{cc} A_{tot} & B_{tot}\\ C_{tot} & D_{tot} \end{array}\right]=\left[\begin{array}{cc} 1 & Z_{COP}\\ 0 & 1 \end{array}\right]\left[\begin{array}{cc} 1 & 0\\ Y_{C_{MetaS\_sh}} & 1 \end{array}\right]\left[\begin{array}{cc} 1 & Z_{L_{MetaS\_se}}\\ 0 & 1 \end{array}\right]\left[\begin{array}{cc} 1 & 0\\ Y_{L_{MetaS\_sh}} & 1 \end{array}\right]\left[\begin{array}{cc} 1 & Z_{B.C.}\\ 0 & 1 \end{array}\right]$$

and

(7) $$a_{tot}=1+\left(Z_{COP}\times Y_{C_{MetaS\_sh}}\right)+\left\{\left(1+\left(Z_{COP}\times Y_{C_{MetaS\_sh}}\right)\right)Z_{L_{MetaS\_se}}+Z_{COP}\right\}Y_{L_{MetaS\_sh}}$$

(8) $$b_{tot}=\left(1+\left(Z_{COP}\times Y_{C_{MetaS\_sh}}\right)\right)Z_{L_{MetaS\_se}}+Z_{COP}$$

(9) $$c_{tot}=Y_{C_{MetaS\_sh}}+\left(Y_{C_{MetaS\_sh}}\times Z_{L_{MetaS\_se}}\right)Y_{L_{MetaS\_sh}}$$

(10) $$d_{tot}=\left(Y_{C_{MetaS\_sh}}\times Z_{L_{MetaS\_se}}\right)+1$$

(11) $$A_{tot}=a_{tot}$$

(12) $$B_{tot}=a_{tot}\times Z_{B.C.}+b_{tot}$$

(13) $$C_{tot}=c_{tot}$$

(14) $$D_{tot}=c_{tot}\times Z_{B.C.}+d_{tot}$$

with $Z_{COP}=-j\frac{1}{\omega C_{COP}},$ $Y_{C_{MetaS\_sh}}=j\omega C_{MetaS\_sh}$, $Z_{L_{MetaS\_se}}=j\omega L_{MetaS\_se}$ and

(15) $$\;\;\;\;Y_{L_{MetaS\_sh}}=-j\frac{1}{\omega L_{MetaS\_sh}}$$

The formulation of Equations (1) to (14) is based on cascading ABCD parameters of neighboring blocks in a circuit. These parameters are adopted to $\Gamma_{}$ through Z-parameters. These parameters in the circuit model analyses are converted to S-parameters, showing the effects of *C*_COP_ of the initial circuit model and *C*_MetaS_sh_, *L*_MetaS_se_ and *L*_MetaS_sh_ of the final circuit model compared to their corresponding measured data. In Figure 10g, as to *C*_COP_, the dashed line as the circuit analysis and the dotted line as its measurement version have dips at similar frequencies, though the levels are different, which is attributed from loss in real implementation. With the final circuit, the solid lines (circular dots and square dots) present improved impedance, matching with discrepancy due to simple circuit modeling. Lastly, the meta-surface combined array sensor is differentiated from other dielectric constant extraction approaches through comparison.

The different measurement techniques are gone over from the viewpoints of the type of configuration, size, frequency, object materials, termination conditions and whether to contact the MUT or not, expressed in Table 3. First of all, only the proposed method aims at a 5G frequency-band and a smart phone-gear, while others do not. This is related to the feature of the novel approach that it deals with thin materials undoubtedly employable for the handset components. If asked what drawbacks the suggested method has, since it counts on meta-surface based impedance matching and specific usages, the accuracy of material characterization will be potentially degraded as characterization bandwidth becomes very broad and the loss tangent exceeds the range between 0.0001 and 0.02 to be of thick and lossy materials. Aside from this derogatory likelihood, as a small sensing device, the proposed method is distinguished from others in that it pursues impedance matching over a specific frequency-band between the probe and the MUT film and devises a novel meta-surface patterned on the COP film, textile or other FPCBs for the objective.

## 6. Conclusions

For the purpose of finding out the permittivity of COP film as a thin material adopted in a millimeter-wave wireless portable device, a new planar sensor is suggested. To enhance the sensitivity of the sensor and make the shape conformal to the surface of layered media of the 5G handset back-cover, the sensor takes the form of a resonator array adequate to TM-mode RF signal excitation and efficient enough to detect the resultant EM-fields from the MUT. In order to conduct accurate *ε*_r_ estimation of the COP film, mismatch from boundaries between the sensor through the COP layer to the back-cover (which deteriorates the quality of the process) is suppressed by designing a meta-surface contacting both the COP film and the back-cover. The impedance matching helps the workflow to get the dielectric constant of the MUT linked with the assumption of the value during impedance matching for low S_11_. As a result, the *ε*_r_ of the COP film proves to be 2.3. To back up the theoretical work of the array sensor and meta-surface, the 5G back-cover (including the fractal cells of the meta-materials and resonator array) was manufactured and the electrical functions were tested. The EM-simulated data (such as impedance) were in good agreement with the measured data. This enables us to know that the relative permittivity evaluated from the measurement becomes almost the same as that in the EM-field analysis. The circuit modelling was also conducted to understand the effects of the constituent layers, and the analysis data were compared with the measured data.

## Figures and Tables

**Table 1:** Features of the conventional dielectric–constant finding methods.

Ref. #	Type	Wide or Narrow Bandwidth	State of the MUT	Analysis Method	Note
[3,5]	Coaxial	Wide	Liquid	Modal analysis	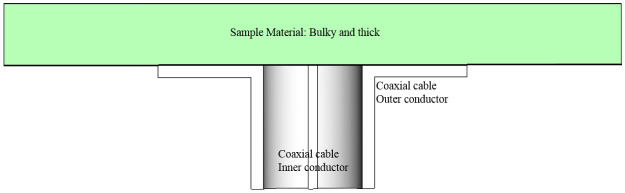
[4]	Coaxial	Wide	Liquid	Cole–Cole plot	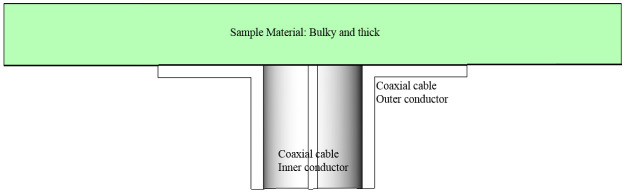
[6]	Coaxial	Wide	Solid	Modal analysis	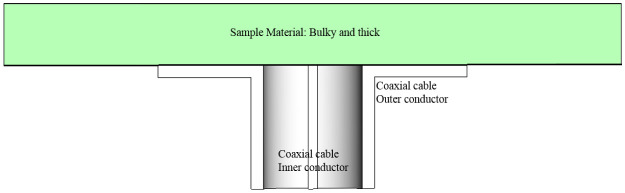
[7,8]	Cavity	Narrow	Liquid	Modal analysis	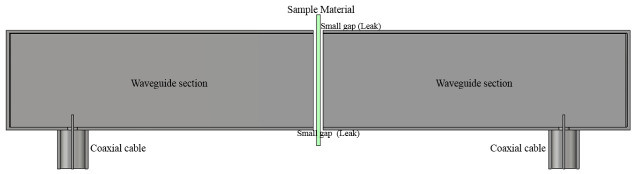
[9,10,11]	Cavity	Narrow	Solid	Modal analysis	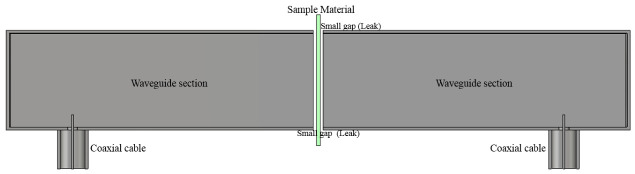
[12,13,14,15,16]	Free space	Wide	Solid	Scattering Parameters	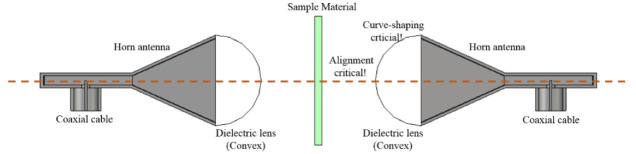

**Table 2:** The physical dimensions of a latest smart phone.

Items	Dimensions
Width	71.52 mm
Length	154.28 mm
Height	2.0 mm
Curvature (Long side)	2.0 mm
Curvature (Corners)	5.0 mm

**Table 3:** Comparison of the proposed method with the schemes from the references.

Ref. #	Type	Size	Freq.	MUT	Termination Condition	Note
[4]	Coaxial	Small, Bulky	3 GHz	Deionized water	Unmatched	Contact
[6]	Coaxial	Small, Bulky	20 GHz	Dissolved Solid	Unmatched	Contact
[7]	Cavity	Mid, Bulky	12 GHz	Liquid	Unmatched	Contact
[9]	Cavity	Mid, Bulky	5.8 GHz	Nano Ferrites	Unmatched	Contact
[12]	Free-space	Biggest	75 GHz	Dielectric	N/A	Distant
Proposed	Coaxial	Small, Thin	28 GHz	Dielectric and Film	Matched	Contact

## Data Availability

Not applicable.
